# Supplementary material for: Porcine Teschovirus 2 3Cpro Evades Host Antiviral Innate Immunity by Inhibiting the IFN-β Signaling Pathway
Source: Microorganisms. 2025 May 26;13(6):1209. doi: 10.3390/microorganisms13061209 (PMC12195036; doi:10.3390/microorganisms13061209)
Supplement: Supplementary file 1 [file microorganisms-13-01209-s001.zip › microorganisms-3586164-supplementary.docx]

**Figure legends**


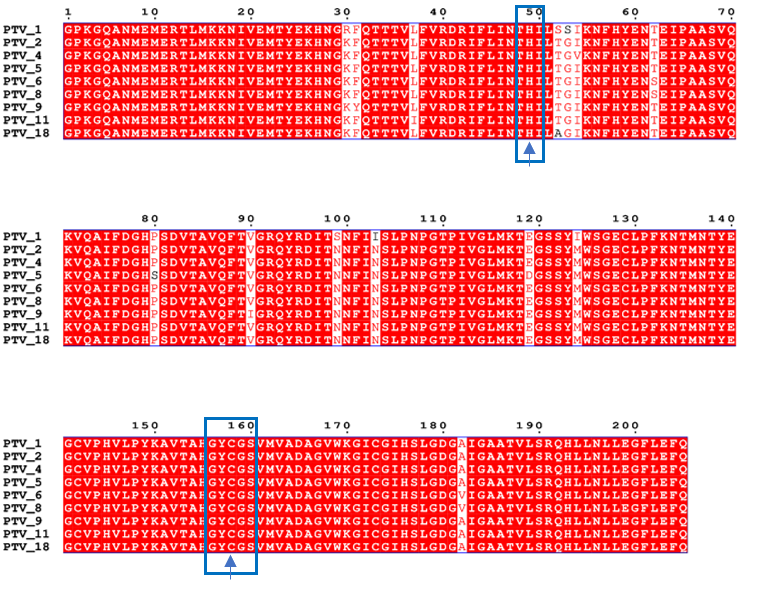


**Figure S1. Cross-genotypic sequence alignment.**

Multiple sequence alignment of 3C protease sequences across different PTV genotypes revealed that the catalytic residues H49 and C158 are conserved in PTV 3C^pro^. The 3C^pro^ sequences used for comparative analysis included PTV genotypes with the following NCBI accession numbers: PTV 1 (NP_740358), PTV 2 (OM281048), PTV 4 (ATL75301.1), PTV 5 (OR962090), PTV 6 (QWS67609.1), PTV 8 (ACT65996.2), PTV 9 (ATL75278.1), PTV 11 (ATL75280.1), and PTV 18 (WVD73044.1). The sequence alignment figure was generated using ESPript 3.0.
